# Complete genome sequence of *Mesorhizobium australicum* type strain (WSM2073^T^)

**DOI:** 10.4056/sigs.4568282

**Published:** 2013-12-15

**Authors:** Wayne Reeve, Kemanthi Nandasena, Ron Yates, Ravi Tiwari, Graham O’Hara, Mohamed Ninawi, Wei Gu, Lynne Goodwin, Chris Detter, Roxanne Tapia, Cliff Han, Alex Copeland, Konstantinos Liolios, Amy Chen, Victor Markowitz, Amrita Pati, Konstantinos Mavromatis, Tanja Woyke, Nikos Kyrpides, Natalia Ivanova, John Howieson

**Affiliations:** 1 Centre for Rhizobium Studies, Murdoch University, Western Australia, Australia; 2 Los Alamos National Laboratory, Bioscience Division, Los Alamos, New Mexico, USA; 3 DOE Joint Genome Institute, Walnut Creek, California, USA; 4 Biological Data Management and Technology Center, Lawrence Berkeley National Laboratory, Berkeley, California, USA; 5 Department of Agriculture and Food, Western Australia, Australia

**Keywords:** root-nodule bacteria, nitrogen fixation, evolution, lateral transfer of genes, integrative and conjugative elements, symbiosis, *Alphaproteobacteria*

## Abstract

*Mesorhizobium australicum* strain WSM2073^T^ was isolated from root nodules on the pasture legume *Biserrula pelecinus* growing in Australia in 2000. This aerobic, motile, gram negative, non-spore-forming rod is poorly effective in N_2_ fixation on *B. pelecinus* and has gained the ability to nodulate *B. pelecinus* following *in situ* lateral transfer of a symbiosis island from the original inoculant strain for this legume, *Mesorhizobium ciceri* bv. *biserrulae* WSM1271. We describe that the genome size of *M. australicum* strain WSM2073^T^ is 6,200,534 bp encoding 6,013 protein-coding genes and 67 RNA-only encoding genes. This genome does not contain any plasmids but has a 455.7 kb genomic island from *Mesorhizobium ciceri* bv. *biserrulae* WSM1271 that has been integrated into a phenylalanine-tRNA gene.

## Introduction

Biological nitrogen fixation (BNF) contributes substantially to the productivity of sustainable agriculture around the world and approximately 80% of biologically fixed nitrogen (N) is estimated to be contributed by the symbiotic association between root nodule bacteria (RNB) and leguminous plants [1]. This process of symbiotic nitrogen fixation (SNF) enables 175 million tons of atmospheric nitrogen (N_2_) to be fixed each year into a plant available form. SNF therefore reduces the need to apply fertilizer to provide bioavailable nitrogen, decreases greenhouse gas emissions derived from fertilizer manufacture, alleviates chemical leaching into the environment from the over application of fertilizer, and substantially enhances soil nitrogen for crop and animal production [2-4]. Because of substantial SNF benefits, considerable effort has been devoted to sourcing legumes from different geographical locations to improve legume productivity in different agricultural settings [3].

The Mediterranean legume *Biserrula pelecinus* L. is one of only three deep rooted annual legume species widely used in commerce with the potential to reduce the development of dryland salinity in Australia and was therefore introduced into Australia in 1994. Native RNB in Australian soil were not capable of nodulating *B. pelecinus* and therefore this host was inoculated with the inoculant strain *Mesorhizobium ciceri* bv. *biserrulae* WSM1271 [5] to obtain an effective symbiosis. Six years after the introduction of this legume into Western Australia, isolates were recovered from root nodules on *B. pelecinus* growing in Northam, Western Australia that were compromised in their nitrogen fixation capacity. The gradual replacement of the inoculant by established strains of RNB that are competitive for nodulation but suboptimal in N_2_ fixation threatens the successful establishment of this new legume in agriculture [6].

One of these poorly effective but competitive strains that was isolated from a nodule of *B. pelecinus* grown in the wheat belt of Western Australia can only fix <40% N_2_ compared to the original inoculant *M. ciceri* bv. *biserrulae* WSM1271. This strain has been designated as WSM2073^T^ (= LMG 24608 = HAMBI 3006) and is now the recognized type strain for the species *Mesorhizobium australicum* [7]. The species name au.stra.li’cum. N.L. neut. adj. australicum is in reference to where this isolate originated from [7] and represents a dominant chromosomal type strain surviving as a soil saprophyte in the Western Australian wheat belt [6,8] that appears to have the capacity to acquire symbiotic genes through horizontal transfer [9].

In this report we present a summary classification and a set of general features for *M. australicum* strain WSM2073^T^ together with the description of the complete genome sequence and its annotation. Here we reveal that a 455.7 Kb genomic island from the inoculant *Mesorhizobium ciceri* bv. *biserrulae* WSM1271 has been horizontally transferred into *M. australicum* strain WSM2073^T^ and integrated into the phenylalanine-tRNA gene.

## Classification and features

**Figure 1:**
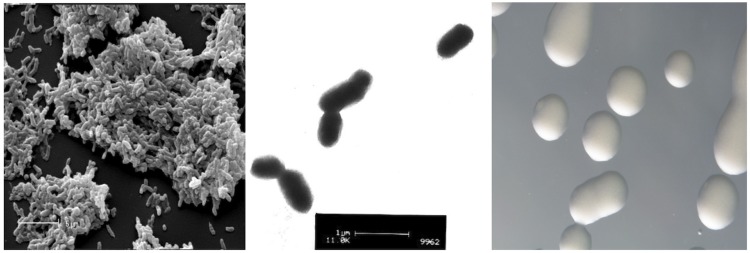
Images of *M. australicum* strain WSM2073^T^ using scanning (Left) and transmission (Center) electron microscopy and the appearance of colony morphology on a solid medium (Right).

*M. australicum* strain WSM2073^T^ is a motile, gram negative, non-spore-forming rod (Figure 1 Left and Center) in the order Rhizobiales of the class Alphaproteobacteria. They are moderately fast growing, forming 2-4 mm diameter colonies within 3-4 days and have a mean generation time of 4 – 6 h when grown in half Lupin Agar (½LA) broth [10] at 28 °C. Colonies on ½LA are white-opaque, slightly domed, moderately mucoid with smooth margins (Figure 1 Right). Strains of this organism are able to tolerate a pH range between 5.5 and 9.0. More information on the carbon source utilization and fatty acid profiles were described before [7]. Minimum Information about a Genome Sequence (MIGS) is given in Table 1.

**Table 1:** Classification and general features of *M. australicum* strain WSM2073^T^ according to the MIGS recommendations [11].

MIGS ID	Property	Term	Evidence code
	Current classification	Domain *Bacteria*	TAS [12]
		Phylum *Proteobacteria*	TAS [13]
		Class *Alphaproteobacteria*	TAS [14,15]
		Order *Rhizobiales*	TAS [15,16]
		Family *Phyllobacteriaceae*	TAS [15,17]
		Genus *Mesorhizobium*	TAS [18]
		Species *Mesorhizobium australicum*	TAS [7]
	
	Gram stain	Negative	TAS [7]
	Cell shape	Rod	TAS [7]
	Motility	Motile	TAS [7]
	Sporulation	Non-sporulating	TAS [19]
	Temperature range	Mesophile	TAS [19]
	Optimum temperature	28°C	TAS [7]
	Salinity	Unknown	NAS
MIGS-22	Oxygen requirement	Aerobic	TAS [19]
	Carbon source	Arabinose, gentibiose, glucose, mannitol & melibiose	TAS [7]
	Energy source	Chemoorganotroph	TAS [19]
MIGS-6	Habitat	Soil, root nodule, host	TAS [7]
MIGS-15	Biotic relationship	Free living, Symbiotic	TAS [7]
MIGS-14	Pathogenicity	None	NAS [19]
	Biosafety level	1	TAS [20]
	Isolation	Root nodule of *Biserrula pelecinus. L*	TAS [7]
MIGS-4	Geographic location	Northam, Western Australia	TAS [6]
MIGS-5	Nodule collection date	August 2000	TAS [6]
MIGS-4.1	Longitude	116.947875	TAS [6]
MIGS-4.2	Latitude	-31.530408	TAS [6]
MIGS-4.3	Depth	10 cm	IDA
MIGS-4.4	Altitude	160 m	IDA

Evidence codes - TAS: Traceable Author Statement (i.e., a direct report exists in the literature); NAS: Non-traceable Author Statement (i.e., not directly observed for the living, isolated sample, but based on a generally accepted property for the species, or anecdotal evidence). These evidence codes are from the Gene Ontology project [21]. If the evidence code is IDA, then the property was directly observed by one of the authors or an expert mentioned in the acknowledgements.

**Figure 2:**
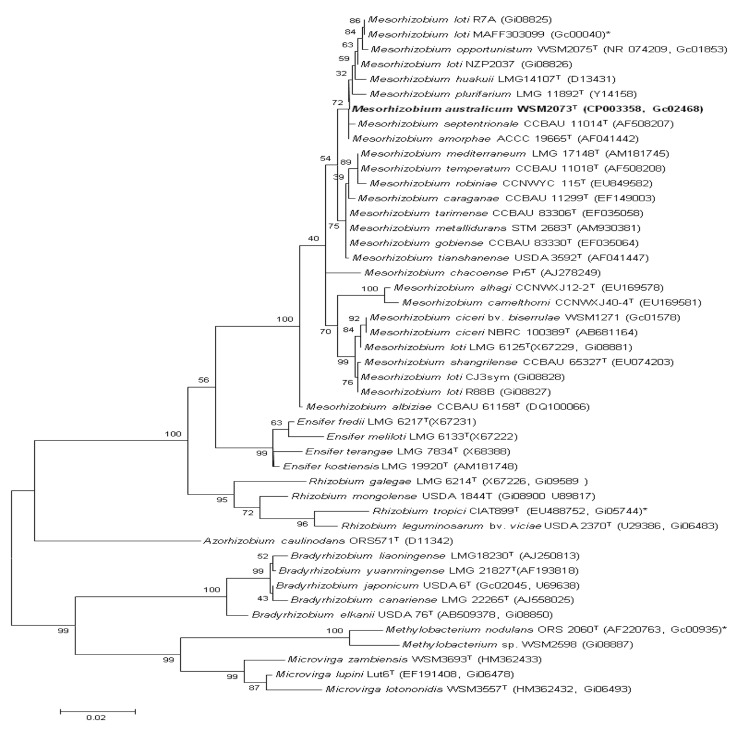
Phylogenetic tree showing the relationships of *M. australicum* strain WSM2073^T^ with some of the root nodule bacteria in the order *Rhizobiales* based on aligned sequences of the 16S rRNA gene (1,290 bp internal region). All sites were informative and there were no gap-containing sites. Phylogenetic analyses were performed using MEGA [22]. The tree was built using the Maximum-Likelihood method with the General Time Reversible model. Bootstrap analysis [23] was performed to assess the support of the clusters. Type strains are indicated with a superscript T. Brackets after the strain name contain a DNA database accession number and/or a GOLD ID (beginning with the prefix G) for a sequencing project registered in GOLD [24] Published genomes are indicated with an asterisk.

Figure 2 shows the phylogenetic neighborhood of *M. australicum* strain WSM2073^T^ in a 16S rRNA sequence based tree. This strain clustered in a tight group which included *M. shangrilense*, *M. loti* and *M. ciceri* and had >99% sequence similarity with all four type strains. However, based on a polyphasic taxonomic study we have identified this strain to belong to a new species [7].

### Symbiotaxonomy

*M. australicum* strain WSM2073^T^ has an extremely narrow legume host range for symbiosis only forming partially effective nitrogen-fixing root nodules on *Biserrula pelecinus L* [6]. This strain also nodulates the closely related species *Astragalus membranaceus* but does not nodulate 21 other legume species nodulated by *Mesorhizobium* spp. [6]. Strain WSM2073^T^ has similar highly specific symbiotic nodulation capabilities to *M. ciceri* bv. *biserrulae* WSM1271, but is a poor N-fixer on *B. pelecinus* L.

## Genome sequencing and annotation

### Genome project history

This organism was selected for sequencing on the basis of its environmental and agricultural relevance to issues in global carbon cycling, alternative energy production, and biogeochemical importance, and is part of the Community Sequencing Program at the US Department of Energy Joint Genome Institute (JGI) for projects of relevance to agency missions. The genome project is deposited in the Genomes OnLine Database [24] and the complete genome sequence in GenBank. Sequencing, finishing and annotation were performed by the DOE Joint Genome Institute (JGI). A summary of the project information is shown in Table 2.

**Table 2:** Genome sequencing project information for *Mesorhizobium australicum* strain WSM2073^T^

MIGS ID	Property	Term
MIGS-31	Finishing quality	Finished
MIGS-28	Libraries used	Illumina GAii shotgun library, 454 Titanium standard library and paired end 454 libraries
MIGS-29	Sequencing platforms	Illumina and 454 technologies
MIGS-31.2	Sequencing coverage	454 standard and paired end (28x) and Illumina (2159x); total 2187x
MIGS-30	Assemblers	Newbler v 2.3 and Velvet v 0.7.63, PHRAP SPS-4.24 and CONSED
MIGS-32	Gene calling method	Prodigal v.2.50, GenePrimp
	Genbank ID	CP003358
	Genbank Date of Release	December 28, 2012
	GOLD ID	Gc02468
	NCBI project ID	47287
	Database: IMG	2509276022
	Project relevance	Symbiotic nitrogen fixation, agriculture

### Growth conditions and DNA isolation

*M. australicum* strain WSM2073^T^ was grown to mid logarithmic phase in TY medium (a rich medium) [25] on a gyratory shaker at 28°C. DNA was isolated from 60 mL of cells using a CTAB (Cetyl trimethylammonium bromide) bacterial genomic DNA isolation method.

### Genome sequencing and assembly

The draft genome of *M. australicum* strain WSM2073^T^ was generated at the DOE Joint genome Institute (JGI) using a combination of Illumina [26] and 454 technologies [27]. For this, genome we constructed and sequenced an Illumina GAii shotgun library which generated 10,509,788 reads totaling 378.4 Mb, a 454 Titanium standard library which generated 235,807 reads and paired end 454 libraries with an average insert sizes of 26.3 Kb /10.9 Kb which generated 221,877/139,171 reads totaling 257.0 Mb of 454 data. All general aspects of library construction and sequencing performed in this project can be found at the DOE Joint Genome Institute website. The initial draft assembly contained 14 contigs in 1 scaffold. The 454 Titanium standard data and the 454 paired end data were assembled together with Newbler, version 2.3. The Newbler consensus sequences were computationally shredded into 2 Kb overlapping fake reads (shreds). Illumina sequencing data was assembled with VELVET, version 0.7.63 [28], and the consensus sequences were computationally shredded into 1.5 Kb overlapping fake reads (shreds). We integrated the 454 Newbler consensus shreds, the Illumina VELVET consensus shreds and the read pairs in the 454 paired end library using parallel phrap, version SPS - 4.24 (High Performance Software, LLC). The software Consed [29-31] was used in the following finishing process. Illumina data was used to correct potential base errors and increase consensus quality using the software Polisher developed at JGI (Alla Lapidus, unpublished). Possible mis-assemblies were corrected using gapResolution (Cliff Han, unpublished), Dupfinisher [32], or sequencing cloned bridging PCR fragments with subcloning. Gaps between contigs were closed by editing in Consed, by PCR and by Bubble PCR (J-F Cheng, unpublished) primer walks. A total of 59 additional reactions were necessary to close gaps and to raise the quality of the finished sequence. The total size of the genome is 6,200,534 bp and the final assembly is based on 257 Mb of 454 draft data which provides an average 28× coverage of the genome and 13,385 Mb of Illumina draft data which provides an average 2159× coverage of the genome.

### Genome annotation

Genes were identified using Prodigal [33] as part of the Oak Ridge National Laboratory genome annotation pipeline, followed by a round of manual curation using the JGI GenePrimp pipeline [34]. The predicted CDSs were translated and used to search the National Center for Biotechnology Information (NCBI) non-redundant database, UniProt, TIGRFam, Pfam, PRIAM, KEGG, COG, and InterPro databases. These data sources were combined to assert a product description for each predicted protein. Non-coding genes and miscellaneous features were predicted using tRNAscan-SE [35], RNAMMer [36], Rfam [37], TMHMM [38], and SignalP [39]. Additional gene prediction analyses and functional annotation were performed within the Integrated Microbial Genomes (IMG-ER) platform [40].

## Genome properties

**Figure 3:**
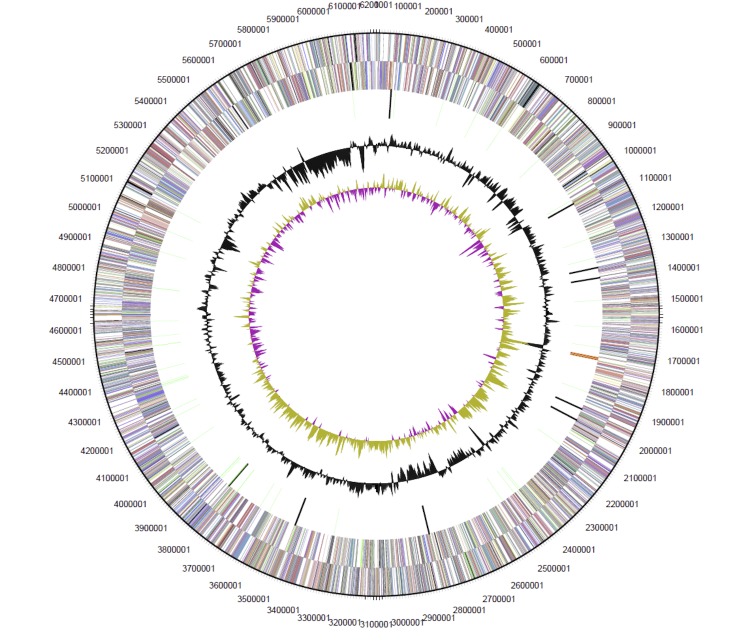
Graphical circular map of the chromosome of *Mesorhizobium australicum* WSM2073^T^. From outside to the center: Genes on forward strand (color by COG categories as denoted by the IMG platform), Genes on reverse strand (color by COG categories), RNA genes (tRNAs green, sRNAs red, other RNAs black), GC content, GC skew.

The genome is 6,200,534 bp long with a 62.84% GC content (Table 3, Figure 3) and comprised of a single chromosome. From all the genes present in the genome, 6,013 were protein coding genes and 67 RNA only encoding genes. Two hundred and twenty one pseudogenes were also identified. The majority of protein coding genes (4,875; 80.18%) were assigned a putative function whilst the remaining protein coding genes were annotated as encoding hypothetical proteins. The distribution of genes into COGs functional categories is presented in Table 4.

**Table 3:** Genome Statistics for *Mesorhizobium australicum* strain WSM2073^T^.

Attribute	Value	% of Total
Genome size (bp)	6,200,534	100
DNA coding region (bp)	5,371,783	86.63
DNA G+C content (bp)	3,896,642	62.84
Number of replicons	1	100
Extrachromosomal elements	0	
Total genes	6,080	100
RNA genes	67	1.1
Protein-coding genes	6,013	98.9
Genes with function prediction	4,875	80.18
Genes assigned to COGs	4,877	80.21
Genes assigned Pfam domains	5,082	83.40
Genes with signal peptides	536	8.82
Genes with transmembrane helices	1,434	23.59
		

**Table 4:** Number of protein coding genes of *Mesorhizobium australicum* WSM2073^T^ associated with the general COG functional categories.

**Code**	**Value**	**%age**	**Description**
J	192	3.56	Translation, ribosomal structure and biogenesis
A	1	0.02	RNA processing and modification
K	450	8.34	Transcription
L	179	3.32	Replication, recombination and repair
B	5	0.09	Chromatin structure and dynamics
D	35	0.65	Cell cycle control, mitosis and meiosis
Y	0	0.00	Nuclear structure
V	60	1.11	Defense mechanisms
T	214	3.96	Signal transduction mechanisms
M	305	5.65	Cell wall/membrane biogenesis
N	42	0.78	Cell motility
Z	0	0.00	Cytoskeleton
W	1	0.02	Extracellular structures
U	115	2.13	Intracellular trafficking and secretion
O	180	3.33	Posttranslational modification, protein turnover, chaperones
C	302	5.59	Energy production conversion
G	511	9.47	Carbohydrate transport and metabolism
E	634	11.75	Amino acid transport metabolism
F	94	1.74	Nucleotide transport and metabolism
H	201	3.72	Coenzyme transport and metabolism
I	216	4.00	Lipid transport and metabolism
P	239	4.43	Inorganic ion transport and metabolism
Q	156	2.89	Secondary metabolite biosynthesis, transport and catabolism
R	699	12.95	General function prediction only
S	567	10.50	Function unknown
-	1203	19.79	Not in COGS
Total	5,748	-	-
